# Institutional Needs

**Published:** 1915-04-10

**Authors:** 


### Institutional Needs.

ARTIFICIAL-LIMB MAKING.

Although it may be stated without fear of contradiction that amputations are to-day less frequently performed than was the case a quarter of a century ago, yet the demand for artificial limbs is by no means an insignificant one, and is, unfortunately, likely to be augmented in the next twelve months. Those familiar with the appliances formerly in vogue cannot fail to be struck with the great improvements which are now embodied in modern artificial limbs. Perhaps it would be more exact to refer to the improvements which should be incorporated if the patient's comfort and usefulness were fully considered, for it is not possible to say even to-day that artificial limbs always present all the best features or conform to the mechanical principles involved in replacing a lost member. As throwing light on what can be done, a survey of Mr. W. R. Grossmith's catalogue affords an instructive lesson. An artificial limb should possess strength, lightness, durability, and natural movement, and should fit accurately. These may be regarded as essential qualities, and it is the careful study of these requirements that has made the work of Mr. Grossmith esteemed by surgeons and patients alike. He has the credit for turning out first-class work from his premises at 110 Strand, W.C., and it must be conceded that his reputation has been maintained. In carrying on a business which was established in Fleet Street over a century and a half ago Mr. Grossmith has ministered appreciably to the comfort of a large number of maimed persons all over the world. His work has been frequently singled out for distinction at exhibitions, but it is understood to be a fact that most of the early improvements in the art of limb-making emanated from the firm which he now conducts. In connection with artificial legs reference may be made to the excellent knee-joint action devised by Mr. Grossmith for producing a light and quick step and taking off the dead weight of the boot. It is often exceedingly difficult to detect nowadays an artificial limb, so well is the natural gait imitated by cunning contrivances to take the place of the normal tendons.

One useful piece of advice is to be found in this catalogue—that is a warning against the suggestion sometimes advanced that those who have recently undergone amputation should commence with a peg-leg. This advice will be endorsed by the surgeons, for the use of a wooden peg-leg produces a stiff and unnatural habit of walking which is not easily overcome. For replacing a lost upper limb there are depicted some satisfactory substitutes which, together with the patent improved attachments for hand instruments, actually afford the maimed individual a very considerable range of activity, and thus greatly reduce his handicap in life. Those who are now working so earnestly to alleviate the discomforts of invalided soldiers will do well, when a case of an amputated limb comes under their care, to seek the advice and the skill of Mr. Grossmith, for in such work as this the highest efficiency is the cheapest, too, in the end.

